# From ions to insulin

**DOI:** 10.7554/eLife.25159

**Published:** 2017-03-09

**Authors:** Voula Kanelis

**Affiliations:** 1 Department of Chemical and Physical Sciences, University of Toronto Mississauga, Mississauga, Canadavoula.kanelis@utoronto.ca; 2 Department of Chemistry, University of Toronto, Toronto, Canada; 3 Department of Cell and Systems Biology, University of Toronto, Toronto, Canada

**Keywords:** ABC transporter, inward rectifier, sulfonylurea receptor, sulfonylurea, None

## Abstract

Electron cryo-microscopy has revealed the three-dimensional structure of a potassium channel that has a central role in regulating the release of insulin from the pancreas.

**Related research article** Martin GM, Yoshioka C, Rex EA, Fay JF, Xie Q, Whorton MR, Chen JZ, Shyng SL. 2017. Cryo-EM structure of the ATP-sensitive potassium channel illuminates mechanisms of assembly and gating. *eLife* **6**:e24149. doi: 10.7554/eLife.24149

The hormone insulin is released from beta cells in the pancreas when the levels of sugar in the blood become too high. High levels of sugar cause the concentration of a molecule called ATP – the energy currency of the cell – to increase inside cells. Ion channels known as K_ATP_ channels sense changes in the levels of ATP to allow potassium ions to move out of beta cells, and thus have a central role in regulating blood sugar levels (Ashcroft, 2005). When ATP levels in the cell are high, the K_ATP_ channels close, and the resulting changes in the electrical excitability of the beta cell membranes lead to an increase in the release of insulin. Likewise, when blood sugar levels decrease, cellular ATP levels also drop, causing the K_ATP_ channels to open so that less insulin is released.

These channels are of considerable medical interest because mutations that affect them can cause diabetes or result in abnormally high levels of insulin, which causes a disease known as hyperinsulinism. Drugs that close K_ATP_ channels to stimulate the release of insulin are widely used to treat type II diabetes, while drugs that open these channels are used to treat mild forms of hyperinsulinism. Now, in eLife, James Chen and Show-Ling Shyng and colleagues at the Oregon Health and Science University – including Gregory Martin as first author – report that they have used electron cryo-microscopy (cryo-EM) to determine the structure of a K_ATP_ channel in the closed state (Martin et al., 2017). In independent work, Ning Gao, Lei Chen and colleagues at Peking University have used cryo-EM to determine the structure of a closed K_ATP_ channel under different conditions (Li et al., 2017).

A pancreatic K_ATP_ channel contains four copies of a pore-forming protein called Kir6.2 and four copies of a regulatory protein called SUR1 (Figure 1A). The SUR1 subunits control the flow of potassium ions through the pore formed by the Kir6.2 subunits. ATP closes K_ATP_ channels by binding to the Kir6.2 subunits. When blood sugar levels are low, another molecule called ADP along with magnesium ions (together known as MgADP) bind to the SUR1 subunits and the channels open. Other factors also regulate K_ATP_ channels, such as the lipid PIP_2_, which opens the channel (Nichols, 2006).

**Figure 1.**
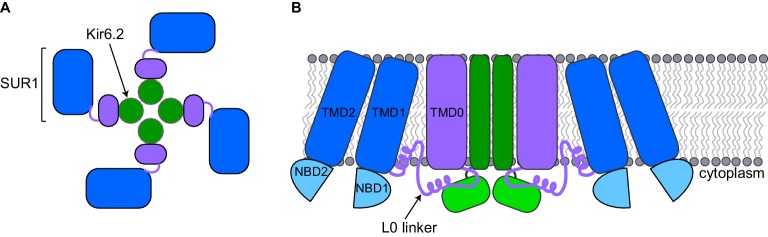
Schematic diagrams of a closed K_ATP_ channel. Each K_ATP_ channel contains four Kir6.2 subunits (green) and four SUR1 subunits. Each SUR1 subunit contains a core (blue), an L0 linker (purple line) and a transmembrane domain called TMD0 (purple). The diagrams are based on the cryo-EM K_ATP_ channel structures by Martin et al. and Li et al. (**A**) Top view of the channel showing the propeller-shaped structure. (**B**) Side view of the channel showing two Kir6.2 subunits and two SUR1 subunits sitting in the cell membrane (gray). The core of SUR1 contains two transmembrane domains (TMD1 and TMD2) and two nucleotide-binding domains (NBD1 and NBD2), while the L0 linker contains two short α-helices. The Kir6.2 subunit is made of two transmembrane helices (dark green rectangle) and two cytoplasmic regions (light green rectangle). Martin et al. and Li et al. found that the center of the K_ATP_ channel propeller structure is formed by the Kir6.2 subunits and the TMD0 of SUR1. The cores of the SUR1 subunits form the propeller blades, but there are unexpected spaces between the cores of adjacent SUR1 subunits that had not been observed in a previously reported structure formed under different conditions (Mikhailov et al., 2005).

The structure reported by Martin et al. was of a pancreatic K_ATP_ channel prepared in the presence of ATP and glibenclamide (a drug that is known to close the channels), while Li et al. solved the structure of the pancreatic K_ATP_ channel prepared in the presence of glibenclamide alone. However, both groups report that the closed channels have similar propeller-shaped structures (Figure 1A).

SUR1 contains a core structure of two membrane-spanning domains and two nucleotide-binding domains (Dean et al., 2001), along with an additional membrane-spanning domain known as TMD0 that is connected to the SUR1 core structure by the L0 linker (Aguilar-Bryan et al., 1995; Figure 1B). The cryo-EM structures show that the SUR1 TMD0 interacts with the Kir6.2 pore. Thus, the cryo-EM structures highlight how TMD0 is important in channel assembly, and explain why this SUR1 domain is a hot spot for mutations that cause hyperinsulinism (Aittoniemi et al., 2009). An unexpected finding is that the SUR1 cores and Kir6.2 do not directly interact, even though the nucleotide-binding domains of SUR1 (which bind to MgADP) are essential for opening the pore (Nichols, 2006) and mutations affecting these domains cause disease (Aittoniemi et al., 2009).

It was known from previous work that Kir6.2 is made of two membrane-spanning helices and two cytoplasmic regions (Figure 1; Hibino et al., 2010). The cryo-EM structures show that ATP, PIP_2_, and glibenclamide bind to regions of the Kir6.2 and SUR1 subunits, and hence explain why both types of subunits affect the action of these molecules. ATP binds to a site between the cytoplasmic regions of adjacent Kir6.2 subunits and a region of the SUR1 L0 linker. PIP_2_ binds to an adjacent site that also involves the Kir6.2 cytoplasmic domains and the SUR1 L0 linker, while glibenclamide binds at a site made by a different region of the L0 linker and the core of SUR1.

The structures highlight the dynamics involved in controlling whether the channel is open or closed. Both teams found that there is a subset of closed channels in which the cytoplasmic domains of Kir6.2 are rotated in a way that may represent a transition toward the open state. Notably, Li et al. show that the inner membrane-spanning helices of two of the Kir6.2 subunits are slightly displaced in this subset of channels, which also possess a bound PIP_2_ molecule.

Previous studies on proteins that structurally resemble SUR1 suggest that, for the channel to open in the presence of MgADP, the SUR1 core has to "untwist" to allow the two nucleotide-binding domains to come into contact (Wilkens, 2015). Further structural studies will be needed to find out whether the twisted SUR1 core in the closed K_ATP_ channel is caused by glibenclamide binding to the channel, and to reveal how other drugs that open K_ATP_ channels work.

Particular regions in the SUR1 transmembrane domains sense conformational changes in the nucleotide-binding domains caused by MgADP binding. Because these regions also bind to the L0 linker, it is possible that conformational changes in the nucleotide-binding domains may alter the structure of the L0 linker. Structural studies of the channels in the open state would shed more light on whether the L0 linker structure changes and may reveal how mutations in Kir6.2 and SUR1 that disrupt the ability of the channel to close can cause diabetes. The exciting structures of K_ATP_ channels reported by Martin et al. and Li et al. lay the foundation for finally answering many of these questions.
